# Confluence of suicide and drug overdose epidemics in young Australian males: common causality?

**DOI:** 10.1186/s12889-018-5875-x

**Published:** 2018-08-03

**Authors:** Richard Taylor, Andrew Page, Alex Wodak, Michael Dudley, Sonali Munot, Stephen Morrell

**Affiliations:** 1 School of Public Health and Community Medicine (SPHCM), Faculty of Medicine, University of New South Wales (UNSW), Kensington Campus, Samuels Building, Level 2, Room 223, Botany St, Gate 11, Randwick (Sydney), 2052 NSW Australia; 2 School of Population Health, Western Sydney University, Sydney, Australia; 3 Emeritus Consultant, St Vincent’s Hospital, Darlinghurst, Sydney, 2010 NSW Australia; 4 School of Psychiatry, University of NSW, Sydney, Australia; 5 Faculty of Medicine, Macquarie University, Sydney, Australia

**Keywords:** Mortality trends, Young adults, Suicide, Drug overdose deaths, Labour market, Unemployment

## Abstract

**Background:**

Young adult (aged 20–34) males experience higher mortality than females, and in age groups immediately younger and older, and with considerable variation in death rates over time. Trends in mortality and the cause structure of deaths among young adult Australian males over 1979–2011 are investigated, with a focus on suicide and drug overdose.

**Methods:**

Mortality data by age for the period 1979 to 2011 and Australian population figures were obtained from the Australian Bureau of Statistics (ABS). Cause of death was investigated using relevant International Classification of Diseases (ICD) codes, and mortality by cause was examined graphically over time according to various ICD aggregations. Mortality trends were contextualised in relation to labour market changes occurring in Australia from the 1980s to early 2000s.

**Results:**

Although motor vehicle accident (MVA) mortality declined by half between 1980 and 1998 in males, this did not translate into a reduction in total young male mortality because of simultaneous increases in suicide, and drug-related deaths classified as either poisoning (external cause) or drug dependence (mental disorders). When both suicide and drug-related deaths declined concurrently after 1998, total 20–34 year male mortality declined by almost half (46%) over 1998–2011. Declines in external cause mortality accounted for 63% of the total mortality decline in 20–34 year males over 1998–2011. The close temporal coincidence (statistically significant) of increases and declines in suicide and drug-related deaths over a decade suggests related causality.

**Conclusions:**

The coincidence of young male suicide and drug overdose mortality epidemics over the study period (excess deaths: 5000) suggest related causality such as exposure to common factors, including the labour market liberalisation and de-regulation of the 1990s, and deserves further investigation.

## Background

Young adults (20–34yrs) represent a fifth of Australia’s total population, and half of these are males (2.2 million) [1]. This age group comprises people increasingly responsible for their own economic support who may either be employed or seeking work after leaving school, or in education or training (part or full-time) and employed or seeking work concurrently. While some of the unemployed may receive partial support from their parents or partner, others will be either full-time students or not seeking work (‘not in the workforce’). They are a group passing through these important complex life transitions with weakening of the support system they experienced during their dependent younger years [2–4]. The profile of young adults also includes risk taking and experimentation, especially in young males [5], and rates of hospitalisation from risky drinking, illicit drug use and dangerous driving have been noted to be higher than other age groups [6].

Young adult males experience higher mortality than ages immediately younger and older, and this excess mortality has been mostly attributed to external and often preventable causes [7], including motor vehicle accidents (MVA), suicide, homicide, and drug overdose [6, 8, 9]. Demographers have noted an ‘accident (or trauma) hump (or bulge)’ in the (log) mortality curve by age, beginning in adolescence and continuing through young adulthood to the mid 30s [10–12]. This observation is probably better labelled a ‘young adult hump (or bulge)’, without mention of trauma, since in previous times (19th and early 20th Century) in the now developed countries [13], and currently in some developing countries, it is a consequence of infectious diseases (especially tuberculosis, and latterly HIV), and causes related to childbirth in females.

Mortality monitoring in Australia [14] has shown higher age-specific death rates in young adult males compared with females since at least 1940 [13, 15]. While mortality in younger Australians (age 12–24 years) halved between 1987–2007, with the male death rate twice that of females [6], mortality rate trends in 25–44 year-olds (‘parent age’) have been relatively stable during the last two decades, with males still experiencing significantly higher rates than females throughout the period [13]. The 20–34 year age group is infrequently selected for detailed examination in routine mortality analysis, however, this age group is selected for the present study after scrutiny of male age-specific death rates during the epidemics of young adult deaths over the study period.

This investigation examines in detail secular trends in young male mortality (20–34 years) in Australia over three decades, 1979–2011, including the sustained decline since 1998, with a focus on the changes in cause structure of mortality that may explain these trends. The study is restricted to males because death rates are at least twice those in females of the same age, and male death rates are characterised by significant fluctuation over time [16]. The study focuses on the 20–34 year age group since this encompasses young adults who have left school, and are affected by changes in the labour market, even if they are also in tertiary or further, post-school education. This age group also captures the experience of the birth cohort most affected by the epidemics of suicide and drug overdose mortality in Australian males in the 1990s period, the first generation of young Australians exposed to the labour market deregulation [16].

## Methods

### Data

De-identified unit record mortality data covering deaths registered in 1979–2012 for each State and Territory were obtained from the Australian Bureau of Statistics (ABS). As numerous deaths occurring in a given year are registered in the following year, especially for external causes, deaths occurring in 2012 were excluded as some of these would have been registered in 2013. Consequently, the study period encompassed deaths occurring in 1979–2011. Cause of death was classified by the ABS according to the standard International Classification of Diseases (ICD), ninth revision (ICD–9), for deaths registered from 1979 to 1996, and by the tenth revision (ICD–10) from 1997 onwards [17, 18]. All ICD–9 codes were converted to ICD–10 prior to analysis (Table 1). Australian annual resident population denominators were obtained from ABS population estimates, derived from quinquennial Census data, for calculating age-specific mortality rates (per 100,000) for 20–34 year males by 5–year age group for each calendar year. The Australian population at 30 June 2001 was chosen as the standard population to calculate directly age-standardised mortality rates for the 20–34 year age bracket [19, 20].

**Table 1:** ICD–10 chapters and individual mortality causes within aggregated chapters used in this analysis

Chapter and scope: Individual or aggregated categories and ICD–10 codes
Chapter XX: External Causes - Injury and poisoning
Motor-vehicle accidents (MVA), suicide and non-intentional drug
overdose
Excluding: homicide, war, legal intervention, medical or surgical
complications, unknown intent, and all other accidents
MVA
MVA: V01–Y89, Y850 (sequelae transport accident)
Drug-related deaths (not coded as intentional)
Accidental Drug Poisoning/Overdose: X40–X44
Accidental drug poisoning by heroin: ICD–10 code X42
All other accidental deaths (not included)
Water and air transport accidents
Falls, mechanical, drowning, fire, electricity/radiation, heat/cold, etc.
(except X40–X44)
Death from intentional self-harm
Suicide: X60–X84, Y870 (sequelae intentional self-harm)
Hanging/Strangulation/Suffocation: X70
Self-inflicted poisoning from Gases, including Motor Vehicle Exhaust
Gases (MVEG): X67
Firearm use (self-inflicted): X72–X74
Self-inflicted Poisoning from Solids/Liquids: X60–X66, X68–X69
Self-inflicted Poisoning from drugs: X60–X64
Chapter V: Mental and Behavioural disorders
ICD–10 codes (drug overdose, use of one or more psychoactive
substances)
F11: opioids, F12: cannabinoids, F14: cocaine; F16: hallucinogens;
F19: other and multiple psychoactive substances
Grouped with drug-related accidental deaths
Excluding: sedatives/hypnotics, alcohol, tobacco, caffeine, volatile
solvents

### Analysis

Based on ICD mortality coding [9, 18], an analysis of ICD chapters was conducted to determine the categories where the majority of deaths occurred among males 20–34 years. Causes within ICD chapters were then aggregated into meaningful categories according to previous and present conventions, and following consultation between co-authors.

Injuries and Poisoning (External Causes) have consistently accounted for about two-thirds of the male deaths in this age group in Australia over 1979–2009 [6, 8]. The causes contributing to the majority of deaths in this Chapter are motor-vehicle accidents (MVA), suicide and non-intentional drug overdose (Table 1). Suicide data are examined as a separate category by means including: Hanging, Strangulation, Suffocation; Self-inflicted poisoning from Gases including Motor Vehicle Exhaust Gases (MVEG); Firearms; and Self-inflicted Poisoning from Solids or Liquids, mostly (80%) due to drugs.

Mortality data for all other ICD chapters (non-external or natural causes) were also examined, and secular trends for the main categories contributing to mortality among young adult males were analysed as: HIV, neoplasms, mental and behavioural disorders, diseases of the circulatory, respiratory and digestive systems, and all remaining Chapters and causes. Accidental poisoning by drugs (External cause Chapter), and drug use (overdose) in the Mental and Behavioural Disorders Chapter, were combined to constitute a combined category of drug-related deaths, as has been employed by the ABS when reporting drug-related deaths, and also by other researchers [15, 21]. Purposeful self-inflicted drug overdose classified as suicide by the Coroner remains in the suicide category.

Age-standardised rates for selected and residual categories of mortality were plotted, and time trends examined, with focus on suicide and accidental drug overdose deaths. For the latter, each series was characterised using standard time series methodology [22], and a time series transfer function was fitted to determine the extent of association between suicide and accidental overdose mortality. Absolute and proportional changes in 20–34 year male mortality between 1998, the peak of all-cause mortality in this population, and 2011, by major cause, are calculated, and the proportional contribution of each to total mortality reduction over the period is estimated. For data manipulation and statistical analyses we employed SAS software version 9.4 [SAS Institute, Chicago IL], and used Microsoft Excel version 12 for graph production [Microsoft Office 2010].

## Results

### Mortality trends

**Fig. 1:**
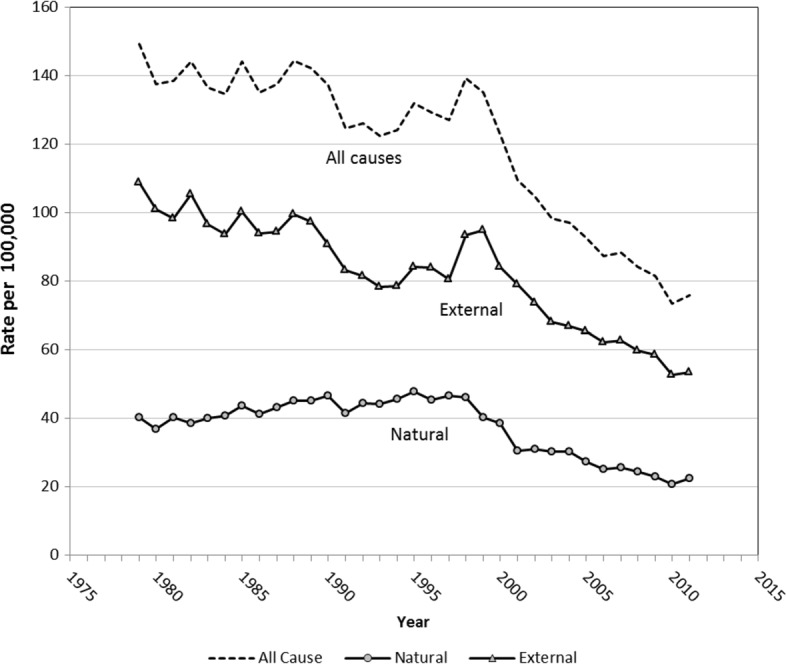
Mortality in 20–34 year males (per 100,000), total and by natural and external causes, Australia 1979–2011

**Fig. 2:**
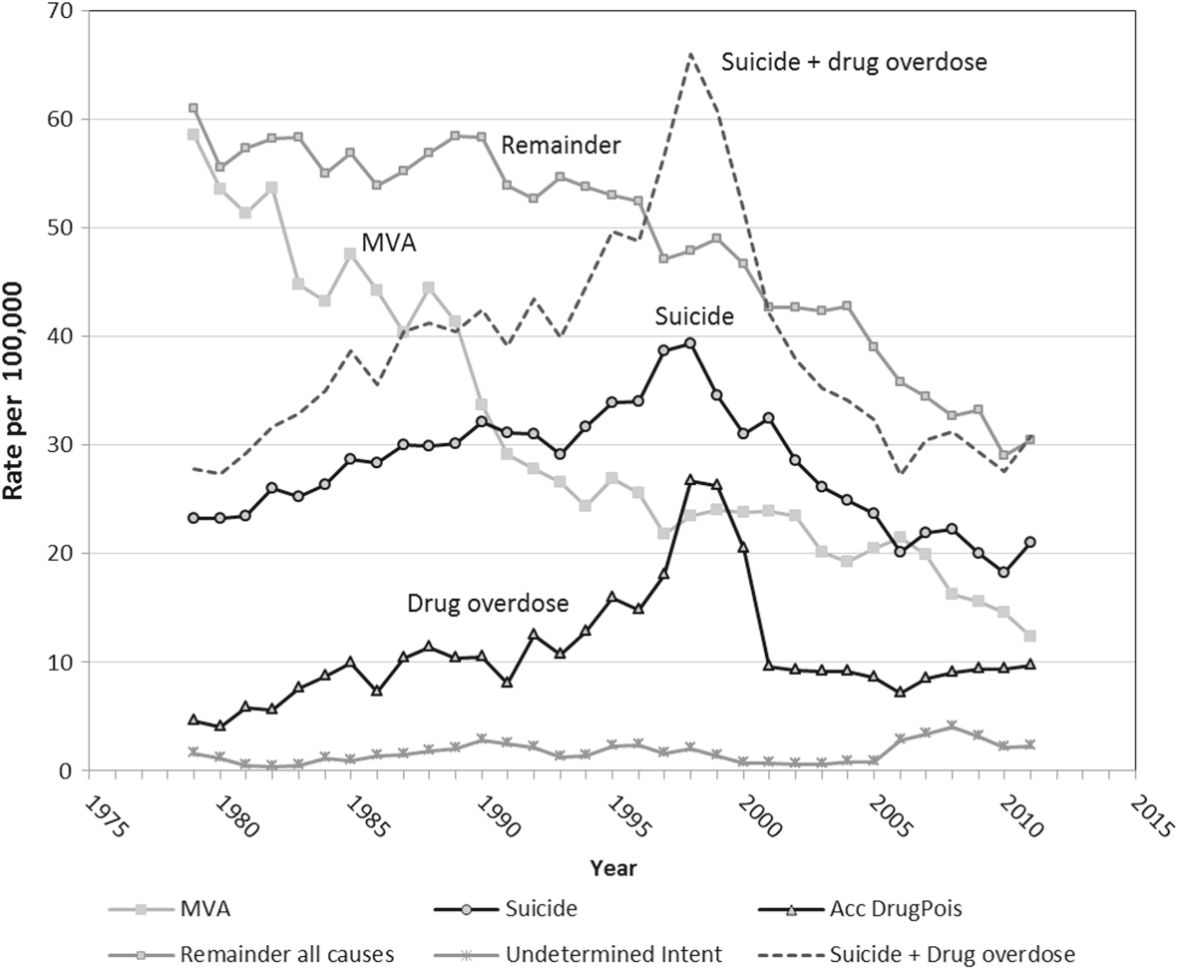
External causes mortality in 20–34 year males (per 100,000), Australia 1979–2011. Drug overdoses include accidental drug overdoses (ICD-10: X40–X44) and death from effects of the drug (ICD–10: F11–F16, F18–F19)

From 1979 to 1989, all-cause mortality in young adult males (20–34yrs) fluctuated around 140 deaths per 10^5^, followed by a decline to 120 deaths/10^5^ during the early 1990s (Fig. 1). A steady rise then ensued, peaking in 1998 at levels (138/10^5^) previously experienced a decade earlier. Since 1998, total young adult male mortality declined to under 80/10^5^ by 2011, a reduction of 46%. Most of the decline has resulted from reductions in external cause mortality (MVA, suicide and drug overdose, Fig. 2). A reduction in natural causes also contributed to this decline.

The mortality decline up to the early 1990s can be attributed to a substantial reduction in MVA deaths which more than halved from 1980 to 1998 (Fig. 2). This reduction did not translate into a reduction in total mortality subsequently, which continued to increase to 1998, because of increases in suicide and drug-related deaths (Figs. 1 and 2). Only when these causes declined (after 1998), along with the continued reduction in MVA deaths, did total young adult mortality resume its decline.

**Fig. 3:**
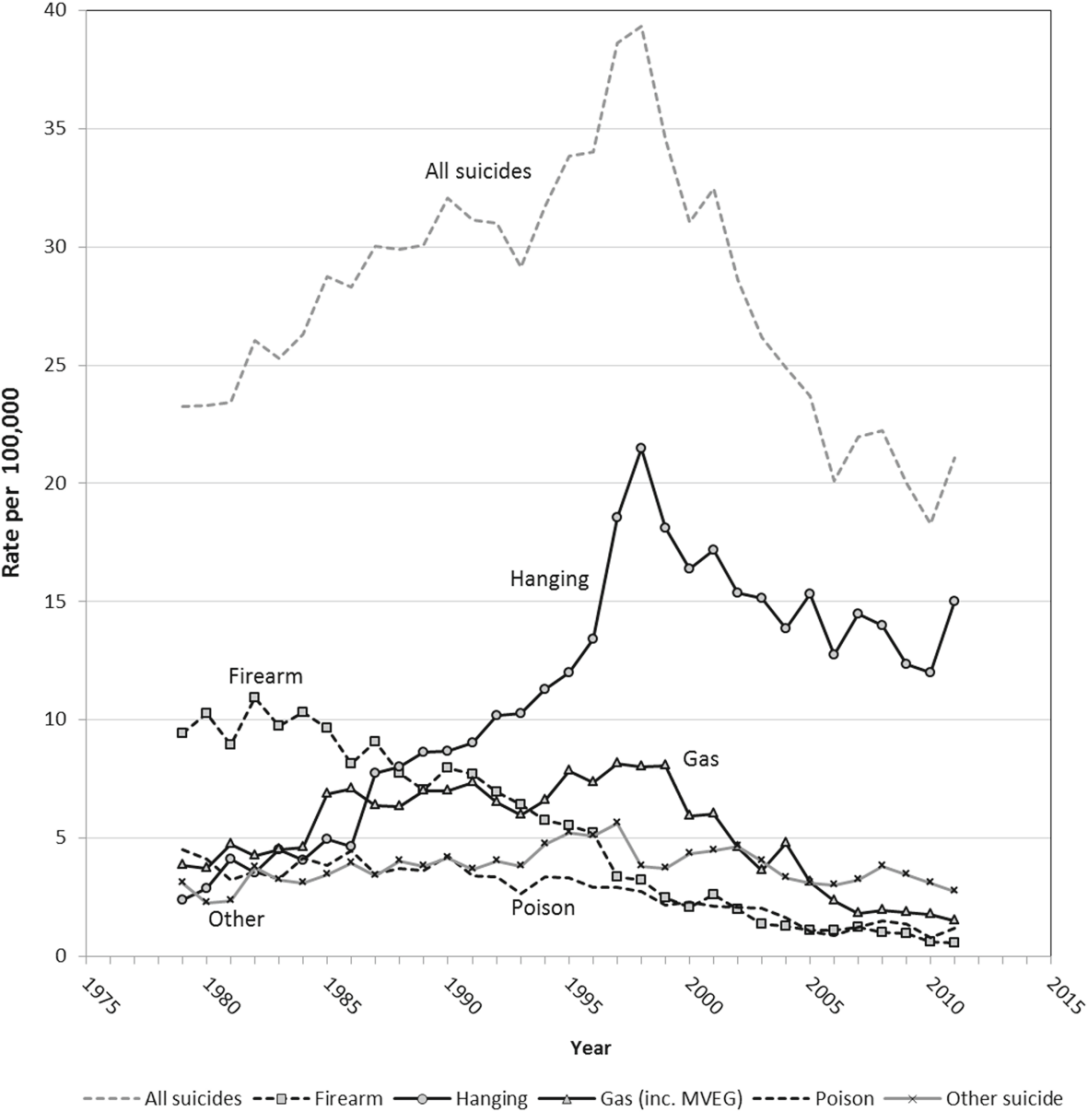
Suicide mortality in 20–34 year males (per 100,000), Australia 1979–2011

Suicide deaths among young adult males almost doubled from 22 deaths per 10^5^ in 1979–81, to a peak of 39 deaths per 10^5^ (*n*=824) in 1998, accounting for over a quarter (28%) of all deaths in the young adult male category (Fig. 3). Since then a remarkably steady decline has occurred, with rates falling back to around 20 per 10^5^ (*n*=513) by 2006, maintained to 2011.

The most common means of male suicide in the early 1980s was by firearm, which has been declining steadily since this period – before and after the 1996 introduction of tighter firearm controls (Fig. 3). Hanging subsequently became by the late 1980s, and remains, the most common suicide method with a trend quite distinct from other means: hanging suicide rates more than doubled from 7–9 per 10^5^ in the late 1980s to 21.5 by 1998, then declined to 12–15 by the first decade of the 21st century. Intentional self-harm from poisonous gases and vapours, mostly Motor Vehicle Exhaust Gas (MVEG) became a commonly employed method for suicide since the mid-1980’s, and by 1995 was the second most frequent method recorded before declining from 2000. The substantial decline in suicide since the 1998 peak to 2011 was mostly from declines in hanging and gassing. The incidence of these two methods combined decreased from 29 per 10^5^ in 1998 to 17 per 10^5^ in 2011 and accounted for 71% of the total suicide decline over 1998–2011 (Table 2). Intentional drug overdose as a means of suicide was minor throughout the period.

**Table 2:** Contributions to 1998–2011 mortality reduction by major cause, Australia, 20–34 year males

Major cause of death	Mortality^a^		Mortality reduction		
	(per 100,000)		Absolute (per 100,000)	Proportional (%)	Proportion of total mortality reduction (%)
	1998	2011			
Suicide	39.3	21.1	18.2	46.4	28.8
Accidental drug poisoning	26.7	9.8	16.9	63.4	26.7
Motor vehicle accident	23.4	12.3	11.1	47.5	17.5
Remaining external	3.8	10.3	(−)6.5	(−)168.6	(−)10.2
All external causes	93.3	53.4	39.9	42.7	62.8
Neoplasm	9.4	7.3	2.1	22.5	3.3
Circulatory disease	7.1	5.4	1.7	24.2	2.7
HIV/AIDS	1.6	0.3	1.3	81.9	2.0
Remaining natural	28.0	9.5	18.5	66.0	29.1
All natural causes	46.0	22.4	23.6	51.3	37.2
All causes	139.3	75.9	63.4	45.5	100
Suicide					Proportion of suicide reduction (%)
Hanging	21.5	15.0	6.4	30.0	35.3
Gassing	8.0	1.5	6.5	81.1	35.7
Firearm	3.2	0.6	2.7	82.3	14.6
Poisoning	2.8	1.2	1.6	57.2	8.6
Other	3.8	2.8	1.0	27.5	5.7

^a^Age standardised (direct) mortality using three 5–year age groups

Aggregated death rates from accidental poisoning by drugs (External cause Chapter), combined with deaths from the Mental and Behavioural Disorders Chapter due to drug use, exceeded 10 per 100,000 in the early 1990s and peaked in 1998–99 at 26/10^5^, then declined steeply by 60% to less than 10 per 10^5^ for 2001–11 (Fig. 2). Drug overdose deaths were noted to be largely due to opiates such as heroin [23].

There trends and peaks (Fig. 2). While the naive correlation coefficient between the accidental poisoning and suicide trends over 1979–2011 was 0.72 suggesting a moderate-to-strong correlation, fitting a time series transfer function between the drug overdose and suicide series to assess their co-incidence yields a numerator regression coefficient (‘moving average’ term) significant at *p*=0.0311, and the denominator regression coefficient (‘autoregressive’ term) was borderline significant at *p*=0.0600. This is evidence for the two series being statistically significantly associated.

**Fig. 4:**
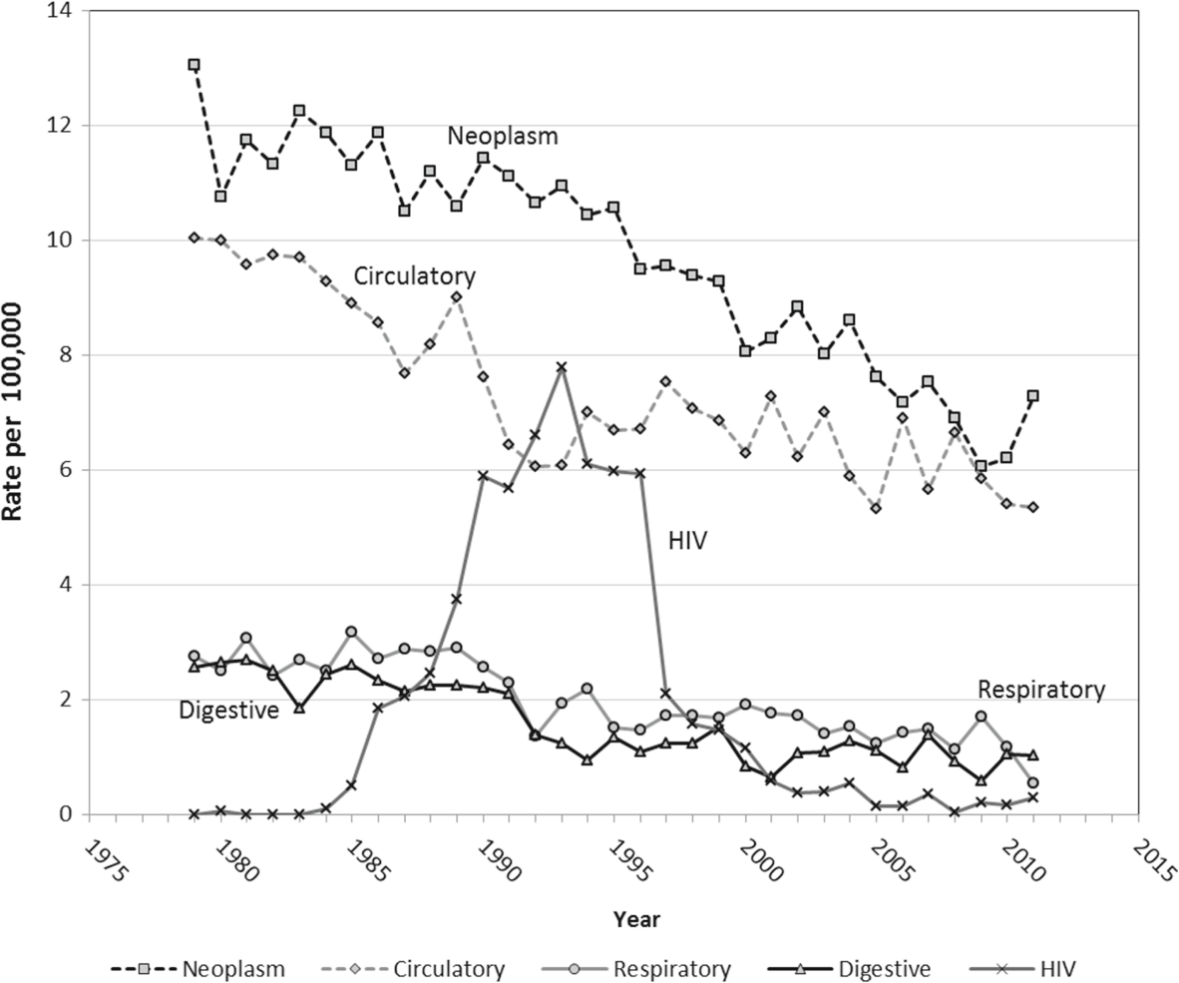
Natural causes mortality in 20–34 year males (per 100,000), Australia 1979–2011

Among natural causes, circulatory system diseases, neoplasms, digestive disorders, and respiratory causes showed a steady decline over this period (1979–2011) (Fig. 4). Deaths due to HIV/AIDS that had steadily risen from 1983, peaked to almost 8 deaths/10^5^ in 1993, then declined to 2 deaths/10^5^ by 1997, and to less than one death per 100,000 over 2000–11.

Deaths due to neoplasms fell after 1995, from 11.0 to 7.5 per 10^5^ by 2011, particularly from lymphoma, chronic myeloid leukaemia, skin cancer, and testicular cancer. Some of these declines were AIDS related. There were also minor declines in mortality from circulatory, digestive and other conditions. Mortality from external causes with undetermined intent (Fig. 2) peaked at 4/10^5^ in 2008, and over 2006–11 was at levels as high or higher than in the 1990s.

### Contributions to 1998–2011 mortality reduction

Mortality reductions from 1998 to 2011 in 20–34 year males occurred across several causes of death. The largest contributors to the total mortality decline were accidental drug poisoning/overdose (27%) and suicide (29%), total 58%, then MVA (18%), followed by various categories of natural causes (Table 2). The contributions to total mortality decline were 63% for external causes and 37% for natural causes, while proportional declines within natural causes was 51% and in external 43%.

By external cause, the contributions to the total suicide decline were largest for hanging (35%) and gassing (36%), while firearms accounted for 15% and poisoning 9%. Within suicide, the largest declines over 1998–2011 were from firearm (82%), gassing (81%) and poisoning (57%). Mortality from accidental drug poisoning/overdose declined proportionally the most (63%), followed by MVA (48%), suicide (46%), and various categories of natural causes.

### Excess deaths from suicide and drug overdose

Estimates are made of the number of suicide and drug overdose deaths exceeding background levels covering the period (1980–2001) of increases and peaks of suicide and accidental drug overdose mortality. If the background levels are taken to be 20 per 10^5^ for suicide (typical during the 1970s for 20–34 year males), and 10 per 10^5^ for accidental drug overdose (see Fig. 2), then 3961 extra suicides and 1038 extra accidental overdose deaths (total =4999) are estimated to have occurred over this period.

## Discussion

This study shows that changes in the cause-of-death structure underlying mortality trends in young adult Australian males (20–34 years) over the 1979–2011 period were substantial. This age and sex group was selected because of the evident epidemic patterns. Mortality declines from MVA, especially in the late 1990s, did not translate into a total (all-cause) mortality decline because of simultaneous increases in deaths from suicide and accidental drug overdose which peaked in the late 1990s, along with a contribution from HIV/AIDS deaths from the mid–1980s to the mid–1990s. From the 1998 peak, a sustained mortality decline of 46% in young adult males occurred over the first decade of the 21st century to 2011. This has been due to continued but slower declines in MVA mortality, substantial declines in suicide and accidental drug overdose, and also significant declines in mortality from HIV/AIDS and neoplasms.

This study is based on routinely collected and coded mortality data and population denominators derived from quinquennial censuses. Australia has complete enumeration of deaths through civil vital registration in each State, and cause is coded centrally for all States from the medical certificate of death by the Australian Bureau of Statistics (ABS). The change from ICD–9 to ICD–10 for mortality coding in 1997 in Australia has not had a significant impact on major causes of deaths in this age group [24], including suicide [24–26]. However, the introduction of ICD–10 coupled with a change from manual to automated coding [27], along with changes in diagnostic fashion, may have affected the relative allocation of drug-related deaths to Mental and Behavioural Disorders, from drug use (overdose), rather than as Accidental Drug Poisoning/Overdose, which is part of the External causes ICD Chapter. Consequently, accidental drug overdose deaths in both categories were combined for analysis in this study, as is also undertaken by the ABS when reporting drug-related deaths [15], and by other researchers [21].

In Australia, all suicide deaths are referred to the coroner for certification. Concerns were raised about the validity of suicide figures in 1997 since major rises in NSW and Victoria were not observed in Queensland, which could indicate interstate data collection differences [28]. An electronic register of coroner cases, the National Coronial Information System (NCIS) commenced operation in 2000. By 2006, visits by ABS officers to coroners’ offices were replaced by using information from the NCIS. Since Coroners’ staff did not always provide complete information to meet the ABS deadline for processing final cause of death data, such as provision of mechanisms of injury, but not intent, ABS officers then assigned codes used for unintentional injury [29]. De Leo et al. (2010) report increasing under-reporting of suicide deaths from 2002 to 2006, estimating between 11% to 16% under-counting in 2004 [30]. From 2006, new rules allowed coronial cases to remain open for up to three years before a determination need be made, after which such cases are relegated to the category of death with undetermined intent. Consequently this has resulted in delays of some years until the final information on suicide deaths is made available [27, 31]. However, these undercount proportions were shown to be too low to affect suicide trends nationally to any significant extent [32]. The delays in establishing cause of death is borne out in the mortality trends for 20–34 year males shown here, where the incidence of external cause mortality of undetermined intent was highest during 2000–11, reaching 4 per 100,000 in 2008, and exceeding the previous highest peak in 1990 by 1.2 per 10^5^. This increase clearly does not account for the 18 per 10^5^ decrease in suicide mortality occurring over 1998–2011 (Table 2) or the >20 per 10^5^ decline over 1998–2008 (Fig. 2).

Some variability exists between different Australian coronial jurisdictions in the classification of suicide, and systematic bias in official suicide statistics may have led to some under-reporting [33–35]. Prior to 2000, the magnitude of this bias does not appear to have significantly affected changes in suicide trends [32, 34, 35]. From 2001, however, misclassification of suicide data by the ABS to other non-intentional causes was reported [27, 30], although this was not sufficient to account for the marked decline in young male suicide since the late 1990s. The study by Page et al. (2010) estimated revised suicide rates under two scenarios of misclassification: (1) adjusting for reported estimates of 9% under-enumeration from misclassification of suicide as an unintentional cause of death; and (2) adjusting for 17% under-enumeration when open cases were also included as suicide [32]. For 1999–2005, officially recorded suicide in 20–34 year males declined by 44% to 22 per 10^5^ in 2005. After adjusting for the first misclassification scenario, suicide rates declined by 38% to 24 per 10^5^; under the second scenario suicide declined by 33% to 26 per 10^5^ [32]. Clearly for the study period, this analysis has shown misclassification issues affect estimates of suicide rates and trends to a minor extent only.

The decline in mortality from HIV in the mid-1990s is likely attributable to preventive campaigns and introduction of combination anti-retroviral therapy, leading to declines in HIV-associated death since the mid 1990s [36].

Declines in cancer mortality in this age group are likely associated with higher survival from incremental treatment improvements in this age group, and treatment for HIV. Cancer mortality in the 15–29 year age group decreased by 2% annually in Australia from 1983 to 2007 [37].

Death rates in 20–34 year Australian males from MVA more than halved between 1980 and 1995, with a slower decline thereafter. Reduction in MVA mortality in Australia since the peak in 1970 has been attributed to successive and cumulative policies including: compulsory use of seat belts (legislated for front seats from 1969); reductions in speed limits and installation of speed cameras; anti-drink driving measures, including random breath testing (introduced during the 1980s in Australian states); targeting locations with higher than average crash rates; and improved roads, safer car designs and Graduated Licensing Systems [38].

Hall and Darke (1998) suggest several proximate factors that may have contributed to the increase in overdose deaths in young adult males in the 1990s, such as increased purity of available opioids, riskier use patterns and polydrug use, although evidence to confirm these hypotheses is limited [39]. A review of accidental and suicidal drug-induced deaths in Australia for 1997–2001 found differing characteristics of each type of death, “suggesting strongly that they are separate issues and thus require different strategies...” [21]. Around 70–80% of drug-induced deaths were classified as accidental. Very few suicide deaths are from illicit drug use: in 2011 these were 1.1 per 10^5^, accounting for 5.2% of all suicides in this age group.

The variation in accidental overdose deaths over time was noted to be consistent with an increase in the availability of heroin in Australia in the mid to late 1990’s, followed by a reduction in availability in 2001 [21, 40–43]. The reduction in heroin supply, reported also by drug users, law enforcement and other agencies, was accompanied by reduced street heroin purity and a doubling of the price [43]. Declining opium production in Burma, which was affected by climatic and political changes and interdiction activities (such as aerial surveillance), has been cited as a reason for the reduction and encouraged a switch to amphetamine trafficking [44–46]. Despite the ‘supply-and-demand’ market scenarios that may explain part of the drug overdose trend, no ultimate underlying socio-economic explanation has been proposed for the increase in young Australian male opioid deaths in the 1990s; and no explanation has been offered as to why the decrease in drug overdose deaths in 1999 in NSW 25–34 year olds was considerably larger than that occurring in 2001 when the supply dropped [47].

In some settings, restriction of availability of means has been shown to be associated with overall declines in suicide rates from all methods. Classic examples include: the changeover from coal gas to natural gas for domestic purposes [48]; and restriction of sedative availability in Australia following liberalisation of sedative prescription policy that led to a suicide epidemic in women during the 1960s [49]. Otherwise, restrictions of certain means have resulted in suicide reductions by those means accompanied by suicide increases by other means (substitution). Firearm-related suicides halved from 10 to 5 per 10^5^ from the 1980s, halving again after tighter gun controls were introduced in 1996 following a mass shooting event in Tasmania [50], with the pre-existing downward trend continuing after the 1996–97 drop (see Fig. 3). Coinciding with the 1996–97 drop in firearm suicide was a steepening of the already sharply rising trend in hanging suicide. Restricted availability of means continues to be regarded as a key strategy for suicide prevention in populations, but this is limited by the reduced means remaining that can feasibly be targeted, since rope or other materials for hanging, and access to many high places for jumping, railway tracks and roads, or other like opportunities cannot easily be restricted. An Australian review of suicide mortality rates and suicide methods over 1998–2007 indicated that the decline in suicide deaths was unlikely to be mediated by the modest reductions in suicidal ideation, from 2.9% to 2.3% as reported in the 1997 and 2007 National Survey of Mental Health and Wellbeing, respectively [51]. The authors attributed the suicide decline rather to the lesser availability of lethal methods, such as reduction in gun availability and increase in catalytic converters in new cars [52].

Evidence for reductions in youth suicide in Australia attributed to reduction in carbon monoxide poisoning accompanying the advent of catalytic converters in motor vehicles comes from a small-area aggregate study of gassing suicide and per capita ownership rates of pre-1986 motor vehicles [53]. Each percentage point of lower pre-1986 vehicle ownership was associated with 6% lower gassing suicide rates. Catalytic converters were introduced as a mandatory requirement in newly manufactured motor vehicles in Australia in 2006.

Declines in hanging and gassing contributed 35% and 36% respectively to the decline in total suicide from the 1998 peak. It is difficult to argue that reduced suicide rates from hanging, which constituted the majority of suicides (50% at the peak in 1998–99), is due to changes in availability of means. Reduction in lethality of MVEG-related suicide attempts, and to a lesser extent hanging, has been claimed to account for approximately half of the 1994–2007 decline in all-age suicide deaths in Australia, based on observations of little or no change in MVEG suicide attempts and increases in attempted suicide by hanging [54]. However, reliable population-wide attempted suicide data are not readily available to separate suicide declines from lowered attempt rates and lowered means availability; and hospital admissions for these causes are not always associated with an intention to die (e.g. ‘auto-erotic asphyxiation’), and not all attempts, regardless of lethal intent, present at a hospital.

Health service changes have also been advocated to reduce suicide, such as increases in funding and access to primary mental health care services with purported suicide prevention capacity [55, 56]. Australian suicide prevention strategies in the 1990s, particularly the National Youth Suicide Prevention Strategy (NYSPS) and the subsequent National Suicide Prevention Strategy (NSPS), attempted to integrate suicide prevention strategies into routine service provision across a spectrum of service providers initially targeting young people and subsequently broadened to the whole population in 1999 [57]. Under the NSPS, first implemented in 1995, a total of $63.5 million, was allocated for the development of national and community-based initiatives for suicide prevention and mental health promotion (between 1999–2006). 150 community-based suicide prevention projects were funded in States and territories, as well as 27 National projects. Despite an apparent secular association between the NYSPS and NSPS and subsequent suicide declines in young adults [16], a more detailed temporo-spatial analysis of the NYSPS initiative showed that it appeared not to make a substantial impact on young male adult suicide trends [57]. While suicide rates did appear to decline significantly in areas with centres receiving suicide prevention funding, the effect was no longer statistically significant after adjusting for area-based measures of socio-economic status (SES), since declines occurred mostly in high SES areas [57], and SES could have acted as an effect modifier. While rates of Medicare-funded mental health service usage are reported to have increased from 3.1 to 6.9% of the whole population over 2006–07 to 2010–11 [58], this more than doubling occurred after most of the suicide fall had occurred and appeared to have little impact on the subsequent suicide trend (cf. Fig. 2). A prior analysis of suicide rates by SES in Australia found the decline in suicide trends among young males, between 1999 and 2004, was confined to medium and high SES areas of residence, while the upward trend continued for young males in the low SES areas [59]. Previous studies in Australia, including young adults, have shown a relationship between suicide (and attempted) and socio-economic status in both aggregate [60–62] and case-control studies [63]; and between unemployment and suicide (and attempted) in both aggregate [64–66] and case-control studies [67].

For suicide in young Australian males, there has been considerable discourse on possible reasons for the decline [48, 49, 51–53, 55–57], particularly the role of restriction of means (firearms, motor vehicle exhaust) and health service interventions, yet there are no suggestions from this discourse as to why the suicide rate increased. None of the supposed reasons for the decline can be invoked (in reverse) to explain the previous rise in the suicide rate, and no ultimate or overarching socio-economic hypothesis has been put forward to account for the observed rise in suicide in 20–34 year males from the 1980s to the peak in 1998. A previous Australian study concluded that the increase in youth suicide (15–24 years) over 1964–1997 was a period effect, from contemporaneous influences, rather than a birth cohort effect from previous experiences [68].

A striking finding from the present study, not previously reported, is the high correlation of secular trends of mortality from accidental drug poisoning/overdose and suicide, confirmed by time series analysis, that suggests a common cause. Suicide increased from long term trends of around 20/10^5^ before 1980, to peak at 40/10^5^ (doubling) by 1997/98; and drug overdose mortality increased from around 10/10^5^ in the 1980s to peak at over 25/10^5^ (2.5 fold increase) by 1998–99. Simultaneous epidemics could be explained by the same period effects.

Concern with young adult unemployment was evident in Australia during the 1980s, and its association with the increasing suicide rate in males in the same age group was noted [64]. From the late 1980s labour market changes were implemented when free market and free trade policies, coupled with weak social protection, were adopted in many countries that relegated the poorest to unemployment, under-employment or lower quality employment, accompanied by higher levels of income inequality and job insecurity [69]. These policies were also enshrined in the Washington Consensus promulgated in 1989 at the end of the Cold War, and directed especially at developing countries [70].

Young workers who first experienced unemployment and under-employment from the effects of economic recession and labour market de-regulation in the 1980s and 1990s, when their situation in the workforce was the least secure due to youth and inexperience, overwhelmingly were forced to accept the changes to their work situations, rendering them even more insecure through individual contracts, casualisation and reduction of other conditions. Young workers entering the workforce after 2000, following the labour market deregulation, experienced the changed situation as the norm, and lesser job security, casual employment contracts and downgraded working conditions had become the (lower) expectation. As anticipated from generational theory, first posited in 1923 [71], the first generation are affected in proportion to the tempo of change, whereas second and subsequent generations entering into an established social and economic architecture will experience less or different effects from such conditions. In Australia, cohort effects are evident by comparisons of suicide rates by age group during the 1990s with the 2000s. The highest suicide rates among males overall during the suicide peak in 1997 were in young adult males aged 15–34 years; a decade later this peak occurred in 25–44 year males, the same cohort [66].

An alternative or compounding (birth) cohort hypothesis is a generational effect for both suicide and drug overdose from potential vulnerabilities acquired by the generation previously affected as children by the increase in marital breakdown following introduction of no-fault divorce in the early 1970s [15]. Those aged 25–34 years in 1990–99 would have been 5–14 years and the first birth cohort exposed to these family and social dislocations. However, this explanation requires further investigation.

The alternative explanation to co-causality for the simultaneous epidemics of suicide and drug overdose is that they are entirely unrelated, and that their co-incidence is by chance, not associated with common social and economic, or other factors.

The existence of a largely deregulated labour market during the global financial crisis (GFC) of 2007–08 is regarded by labour market economists as a key factor in the Australian economy emerging relatively unscathed from the GFC [72]. Significantly higher rates of unemployment were avoided by the ability of the employers to instead increase under-employment by reducing working hours, impose unpaid leave or part-time employment without retrenchment, or transfer workers from full-time to part-time employment from the ‘flexibility’ afforded by labour market deregulation that had previously been implemented, particularly in the 1990s. ABS figures show the proportion of all employed males aged 15–24 years who were in part-time employment has exceeded the unemployment rate in 15–24 year males since April 1987.

While it has been acknowledged that such labour market flexibility did not save the US from the effects of the GFC [72], the other key factor contributing to Australia being relatively unaffected by the GFC was a large government stimulus package and the continuing demand from China for mineral exports. Over the two decades of 1980–2001 covering periods of high unemployment levels, particularly high youth unemployment relative to overall [64], labour market deregulation, and the co-incident increases and peaks of suicide and accidental drug overdose mortality, approximately 5000 young male adult deaths from suicide and accidental drug overdose occurred in excess of the numbers expected from previous background levels.

The purpose of the present study has been to delineate aggregate population-level trends in Australian young male mortality, and to propose plausible explanations. Strengths of this study are the inclusion of the total population of young adult (20–34 years) males over an extended period, and consideration of trends in cause-specific mortality in the context of total mortality, with specific comparison of trends in suicide and drug overdose mortality. The quality of the cause of death data is consistent across the study period, with possible anomalies investigated and shown to be minor. Study weaknesses are those common in cause-of-death analyses from routine statistics, as compared with cohort studies of individuals containing detailed information on putative causes and confounders in both decedents and non-decedents.

## Conclusions

The confluence of suicide and drug overdose epidemics coinciding with labour market deregulation in the first generation of Australian youth to experience such changes may be explained at an individual level by differing reactions to a sudden change in social and economic circumstances for the worse. Some may direct their psychological and emotional reactions inwards and take their own life. Others may react to downturns in life’s prospects by self-medicating with illicit drugs, thus exposing themselves to the risk of unintentional overdose. Whatever the exact mediating factors, the excess deaths from suicide and drug overdose between the 1980s and early 2000s, impacting on the first generation of Australian young men exposed to labour market de-regulation, merits further investigation since it has arguably more cogency than explanations so far proffered for separate epidemics. The price of labour market ‘flexibility’, so lauded by economists for its purported role in Australia’s accommodation to the GFC, may have been 5000 excess deaths in young men.

## Abbreviations

ABS: Australian Bureau of statistics
AIDS: Acquired immune deficiency syndrome
GFC: Global financial crisis
HIV: Human immunodeficiency virus
ICD: International classification of diseases
MVA: Motor vehicle accident
MVEG: Motor vehicle exhaust gas
NCIS: National coronial information system
NSPS: National suicide prevention strategy
NYSPS: National youth suicide prevention strategy
SES: Socio-economic status
